# Partial Genome Sequence of a Novel Reo-Like Virus Detected in Asian Citrus Psyllid (Diaphorina citri) Populations from Florida Citrus Groves

**DOI:** 10.1128/MRA.00563-21

**Published:** 2021-08-26

**Authors:** Kellee Britt, Kristian Stevens, Samantha Gebben, Amit Levy, Maher Al Rwahnih, Ozgur Batuman

**Affiliations:** a Department of Plant Pathology, Southwest Florida Research and Education Center, University of Florida, Immokalee, Florida, USA; b Department of Plant Pathology, University of California-Davis, Davis, California, USA; c Department of Plant Pathology, Citrus Research and Education Center, University of Florida, Lake Alfred, Florida, USA; DOE Joint Genome Institute

## Abstract

This report describes the partial (nearly complete) genome sequence of a novel reo-like virus tentatively named Diaphorina citri Cimodo-like virus. This putative virus has 10 double-stranded RNA segments and was detected in Asian citrus psyllid (Diaphorina citri) populations collected from Florida commercial citrus groves.

## ANNOUNCEMENT

Huanglongbing, the world’s worst citrus disease, is caused by the bacterium “*Candidatus* Liberibacter asiaticus” and vectored by the Asian citrus psyllid (ACP) (Diaphorina citri) (1, 2). Insect-specific viruses (ISVs) can offer potential biological alternatives as lethal and efficient viral vectors toward their respective invertebrate hosts (3). To identify ISVs of Florida ACP populations, high-throughput sequencing (HTS) was conducted on ACPs collected from Florida citrus groves (4). Subsequent bioinformatic analyses revealed the presence of sequences belonging to a novel reo-like virus with consistent closest protein similarity to the Cimodo virus (CMDV), a reovirus isolated from African mosquitoes (5). Viruses in the *Reoviridae* family have been found in many different eukaryotic hosts, including insects, and are composed of 9 to 12 double-stranded RNA segmented genomes (6, 7).

HTS preparation for identification of this novel reo-like virus was previously described by Britt et al. (4). Briefly, total RNAs were extracted using TRIzol reagent (Thermo Fisher Scientific, Waltham, MA, USA), according to the manufacturer’s instructions, from a pool of adult and nymph ACPs (*n* = ∼30) that had been collected from Florida citrus groves in 2020. Total RNAs extracted from the sample were quantified and determined to be of sufficient quality for HTS using a Synergy HTX plate reader (BioTek Instruments, Winooski, VT, USA). An aliquot of the sample was separated and subjected to rRNA depletion and subsequent cDNA library construction using a TruSeq stranded total RNA with Ribo-Zero plant kit (Illumina, San Diego, CA) at the Foundation Plant Services at the University of California-Davis (Davis, CA, USA). The HTS sample was then sequenced on the Illumina NextSeq 500 platform as described previously (8).

The 2,878,296,525 resulting single-end Illumina reads of 75-bp length from the cDNA library were demultiplexed, adapter trimmed, and filtered using Illumina bcl2fastq software v2. The 38,377,287 trimmed and filtered reads were then *de novo* assembled into contigs using SPAdes v3.14 with default parameters (9). The *de novo* assembled contigs were then compared with a July 2020 copy of the GenBank database from the National Center for Biotechnology Information (NCBI) using BLASTn v2.10.1 with a word size of 7 and default parameters.

Ten viral contigs of a putative reo-like virus, tentatively named Diaphorina citri Cimodo-like virus (DcCLV), were identified and were subsequently confirmed back in the HTS sample using reverse transcriptase (RT) PCRs for the positive-sense strand, as well as selected negative-sense strands (Table 1). As an additional confirmation, amplified PCR fragments were Sanger sequenced and showed 90 to 99% identity to corresponding consensus sequences obtained through HTS (Table 1). Putative open reading frames (ORFs) for each segment were identified using NCBI ORF finder and annotated using a BLASTp search of the nonredundant protein sequence database (Table 1). A numbering scheme for DcCLV segments was determined based on the homologous CMDV segment protein and then numerically for more divergent segments (Table 1).

**TABLE 1:** Annotation and characteristics of DcCLV putative proteins in relation to CMDV

Putative DcCLV segment	Length (bp)	GC content (%)	No. of amino acids	Homologous CMDV segment and protein^*a*^	Mapped read coverage of contig (×)	Predicted isoelectric point	BLASTp coverage (%)	BLASTp identity (%)	E value	Strand (+/−) confirmed by RT-PCR and Sanger sequencing
S1	4,084	38.1	1,216	S1, RdRp (accession no. YP_009072449)	68	8.6	99	43	0	+, **−**
S2	3,713	40.2	1,209	S2, hypothetical protein (accession no. YP_009059073)	45	6.0	96	35	0	+
S3	3,243	37.1	1,065	S3, hypothetical protein (accession no. AHF20717)	80	6.6	99	33	5^−169^	+, **−**
S4	2,306	39.0	700	S4, hypothetical protein (accession no. YP_009059075)	66	8.6	99	50	0	+
S5	2,358	38.8	636	S5, hypothetical protein (accession no. AHF20719)	72	5.7	49	28	6^−34^	+
S6	1,840	37.2	522	S6, NTP binding domain protein (accession no. AHF20720)	77	6.2	95	28	1^−52^	+
S7	2,415	35.6	754	S7, hypothetical protein (accession no. AHF20721)	79	5.5	17	35	1^−11^	+
S8	1,636	37.4	489	S8, hypothetical protein (accession no. YP_009059068)	92	4.3	63	25	8^−15^	+
S9	872	39.3	258	S10, hypothetical protein (accession no. YP_009059070)	49	6.6	55	26	5^−3^	+
S10	1,029	39.1	263	S11, hypothetical protein (accession no. YP_009059071)	124	6.8	85	25	9^−10^	+

a RdRp, RNA-dependent RNA polymerase; NTP, nucleoside triphosphate.

In conclusion, the significant but consistent low levels of amino acid identity between DcCLV segments and the corresponding segments of CMDV suggest that DcCLV may belong to an unclassified genus in the reovirus subfamily *Spinareovirinae*, like CMDV.

### Data availability.

DcCLV segment sequences are listed under the following GenBank accession numbers: MZ484733, MZ484734, MZ484735, MZ484736, MZ484737, MZ484738, MZ484739, MZ484740, MZ484741, and MZ484742. The associated read sequence data are publicly available under the SRA accession number SRR14811709.
